# Immediate Loading of Zygomatic Implants Using a Dual Scan Technique

**DOI:** 10.3390/jcm12237464

**Published:** 2023-12-01

**Authors:** Mustafa Gseibat, Valerio Sorrentino, Pablo Sevilla, Jesús Peláez, Maria J. Suarez

**Affiliations:** Department of Conservative Dentistry and Prostheses, Faculty of Odontology, University Complutense of Madrid, 28040 Madrid, Spain; mam@ucm.es (M.G.); valeriso@ucm.es (V.S.); pasevi01@ucm.es (P.S.); mjsuarez@ucm.es (M.J.S.)

**Keywords:** zygomatic implants, immediate loading, stereophotogrammetry, digital impressions, prosthodontics

## Abstract

The immediate loading protocol has become increasingly popular due to the progressive growth in demand for a reduction in treatment times. The possibility of applying this protocol would depend on certain important factors. The application of the digital workflow mentioned in the protocol guarantees rapidity, precision, and esthetics. This report aims to describe a fully digital workflow using a dual scan impression technique to fabricate immediate fixed complete dentures (FCDs) for zygomatic and standard implants. A 58-year-old female patient requested treatment for her severely atrophic maxilla, and four unrehabilitated implants in the mandible. After proper diagnosis and planification, four zygomatic implants and two standard implants were placed. During the surgery, transmucosal abutments were placed on all implants. After suturing, the positions of the implants were recorded using a stereophotogrammetric technique, creating a standard tessellation (STL) file. In the lower arch, the second phase of the surgery was carried out: the transmucosal abutments were placed, and then the implant positions were recorded in the same way. The soft tissues were rescanned after suturing with an intraoral scanner (IOS), and all STL files were aligned to obtain the virtual final models. The pre-design after virtual modifications was aligned with the definitive models. The provisional prostheses were milled and placed after six hours after the surgery, and the definitive prostheses were placed six months after the surgery. The dual scan technique used obtained a precise fit for both the provisional and definitive FCDs. This technique might be an effective and reliable alternative for the fabrication of immediate and definitive screw-retained FCDs in a completely digital workflow. The time taken for scanning and fabrication was reduced, and the clinician’s and patient’s satisfaction were improved.

## 1. Introduction

Zygomatic implants are considered as an alternative option to rehabilitate the atrophic maxilla, with their main advantage being that they can overcome the complications related to grafting techniques and reduce the treatment period. Zygomatic Implants were first introduced by Brånemark in 1998 [1,2]. This method was originally developed for patients who had undergone maxillary resection due to malignancy and who required retention for an obturator. Brånemark developed a specific implant called a zygomatic fixture to provide a fixed solution, even when the conditions for implant insertion were poor in the posterior areas of the maxilla, offering an alternative to bone grafting or sinus lift procedures, which involve quite invasive surgery [3,4]. He considered using the zygomatic bone as an anchorage for prosthetic rehabilitation in hemi-maxillectomy patients, as well as for other defects. The combination of an immediate loading protocol with the zygomatic implants will significantly accelerate the treatment period [5,6]. These implants showed good long-term clinical results, both in immediate and delayed loading protocols, with a survival rate of 86% to 100% for a period of 5 to 18 years, and a failure rate of 3% for a period of 5 to 18 years [7,8,9,10,11,12,13].

Loading protocols have been a subject of discussion since the origin of osseointegration. In 1990, the first study was published, in which it was suggested that implants could be loaded immediately or early in the jaws [14]. Immediate loading can be defined as loading a dental implant immediately or within hours of placement. However, many definitions can be found in the literature. Currently, Esposito et al.’s protocol has been accepted, with immediate loading occurring earlier than one-week, early loading between one week to two months, and conventional loading occurring more than two months after implant placement [15]. The factors influencing the results of the immediate implant loading of a fixed prosthesis are primary stability, implant length, implant design, implant quantity, bone quality and quantity, parafunction, and prosthesis design [16]. Several protocols have been proposed for the treatment of edentulous jaws, mainly in terms of the number of implants be used, their strategic distribution, the use of a provisional prosthesis, and the design of the definitive prosthesis.

Digital technology in dentistry has developed remarkably over the last few years. In this specialty, intraoral and extraoral scanners and stereophotogrammetry and computer-aided design/computer-aided manufacturing (CAD/CAM) technology play a very important role in the development of this digital technology since they are the first steps towards fully computerized dentistry.

The stereophotogrammetric technique in dentistry is considered to be a good alternative to conventional impression techniques [17]. In the case of multiple implants, the photogrammetry technique has several advantages over some other impression techniques, such as precision, rapidity, less complicity, and conformability for both patients and dentists [18,19]. However, clinical studies are limited.

The aim of this study was to describe and evaluate a fully digital workflow using a dual scan impression technique to fabricate immediate fixed complete dentures for zygomatic and standard implants.

## 2. Material and Methods

**Figure 1:**
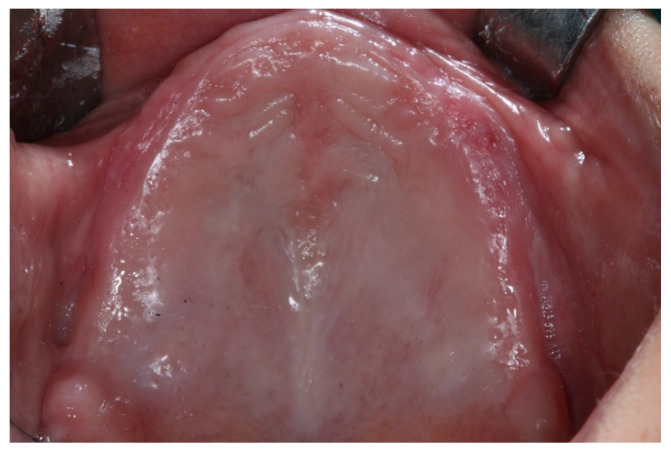
Intraoral preoperative view.

**Figure 2:**
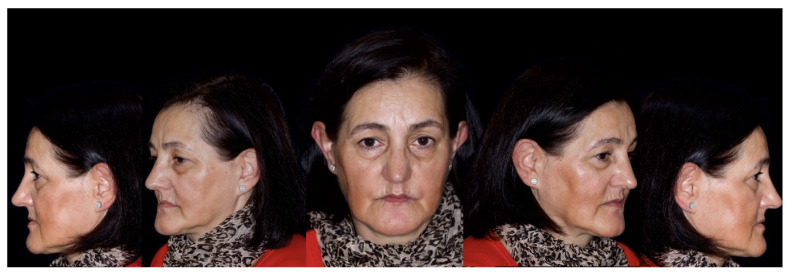
Extraoral aspects of the patient.

A 58-year-old female patient, carrying a maxillary complete denture and a mandibular removable partial denture, was referred to the master’s degree students in Bucofacial Prosthesis and Occlusion (Faculty of Odontology, University Complutense of Madrid, Spain), seeking a fixed prosthodontic rehabilitation in the shortest possible time. Clinical examination, intraoral and extraoral photographs, and esthetic, function, and radiographic analyses (panoramic radiography and Cone Beam Computed Tomography, CBCT) were undertaken (Figure 1 and Figure 2).

Intraoral examination showed atrophic maxilla (class V maxillary alveolar process according to Cawood and Howell’s classification) [20], and the use of a screw-retained fixed complete denture (FCD) supported by four zygomatic implants and two standard implants was planned. In the mandible, four bone-level implants (Brånemark System Mk III TiUnite RP 4 × 10 mm; Nobel Biocare, Zurich, Switzerland) were placed in the interforaminal region, and a screw-retained FCD was also indicated. Furthermore, in the lower arch, two posterior periodontally compromised molars (Teeth number: 36, 46) were found. The treatment plan was discussed with the patient and informed consent was sought and obtained. 

**Figure 3:**
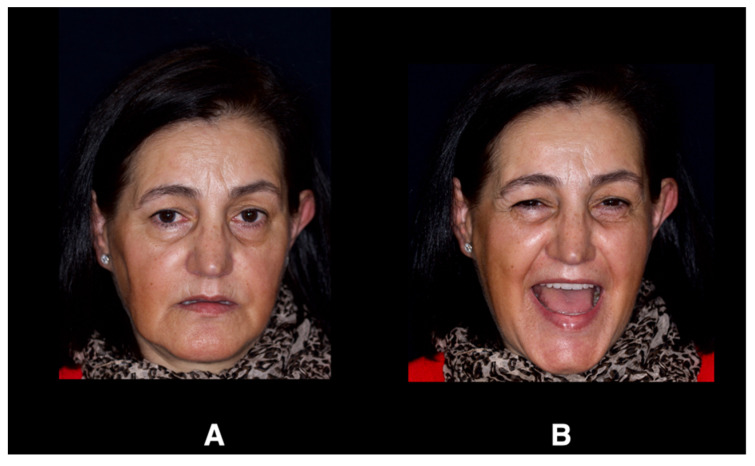
Incisor exposure and esthetic appearance of the patient’s removable prostheses; (**A**) at rest position and (**B**) smile position.

**Figure 4:**
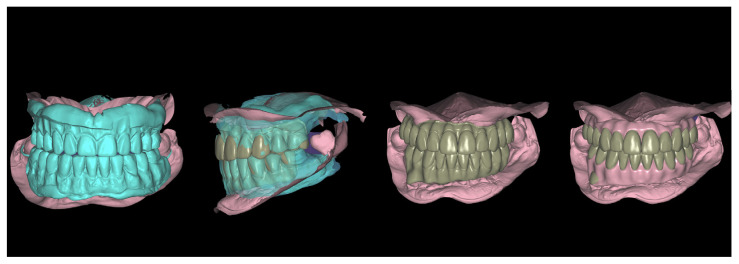
The virtual predesign of the provisional FCDs.

The treatment commenced with scanning the patient’s upper and the lower prostheses using an intraoral scanner (IOS) (Trios 3; 3Shape, Copenhagen, Denmark). The scan information constituted the first STL file. Both prostheses were removed, and another intra oral scan of upper and lower arches was performed to create a second STL file. The vertical dimension was recorded by scanning the buccal surfaces of maxillary and mandibular teeth in centric occlusion. Using CAD/CAM software (Exocad 2.4 Plovdiv; Exocad GmbH, Darmstadt, Germany), the first and second STL files were aligned and matched in the recorded vertical dimension, and the centric occlusion was used to design the immediate FCDs. During the design process, some modifications were carried out on the teeth shape: the incisor teeth length was increased by 1 mm to improve the incisor exposure and esthetic appearance (Figure 3), and the vertical dimension was increased by 0.5 mm to compensate for the increase in incisor teeth length over time. A virtual predesign of the provisional FCDs was obtained (Figure 4). The clinicians conserved the two remaining mandibular molars to use them as landmarks during the process of virtual alignment.

**Figure 5:**
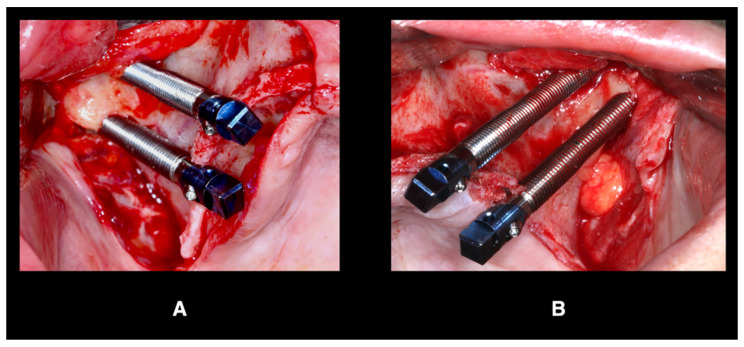
ZAGA type-4 path; (**A**) right side, (**B**) left side.

**Figure 6:**
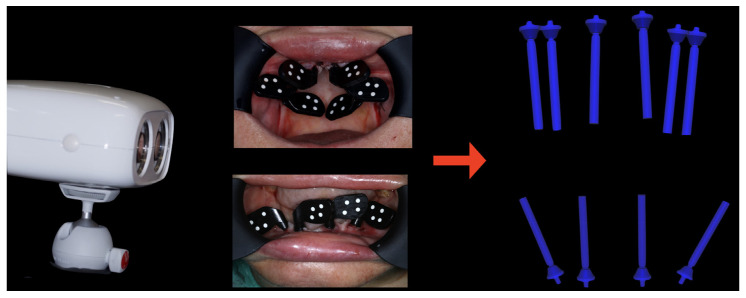
Capturing of implants positions using PIC Camera.

As part of the master’s degree in Oral Surgery and Implants at the same University, four zygomatic implants (Zygomatic HE Implant; Neodent, Curitiba, Brasil), and two bone-level implants (TSVT MTX Implant 3.7 × 10 mm; ZimVie, Palm Beach Gardens, FL, USA) were placed. The zygomatic implants were placed according to the zygomatic anatomy-guided approach (ZAGA type-4 path) (Figure 5) [4]. During the surgery and after implants placement, primary stability of all implants was confirmed, and transmucosal abutments were placed with a 25 Ncm torque. The scan bodies (PIC transfers, PIC Dental, Madrid, Spain) were placed, and the implants positions were recorded using a photogrammetry system (PIC Camera, PIC Dental) creating an STL file (PIC file) (Figure 6). In the lower arch, the second phase of surgery was carried out: the transmucosal abutments were placed and tightened to 30 Ncm, and then the positions of the implants were recorded in the same way creating the second PIC file. The soft tissue was also rescanned to copy the changes that occurred due to the surgery, and then the two compromised lower molars were extracted.

**Figure 7:**
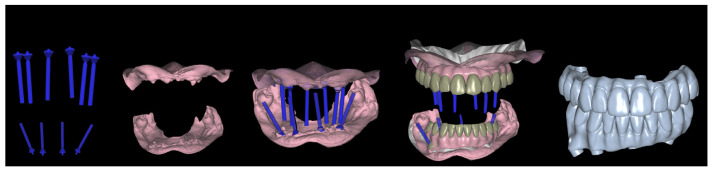
Definitive virtual design of the immediate FCDs.

**Figure 8:**
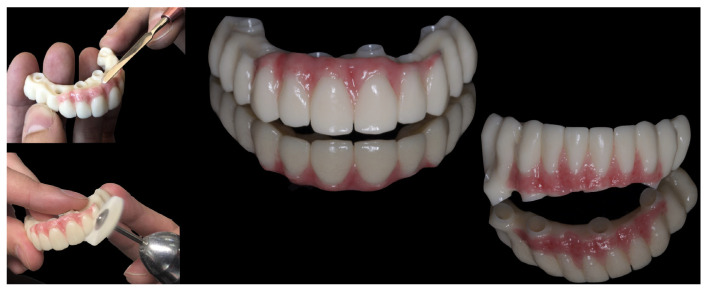
Immediate FCDs.

**Figure 9:**
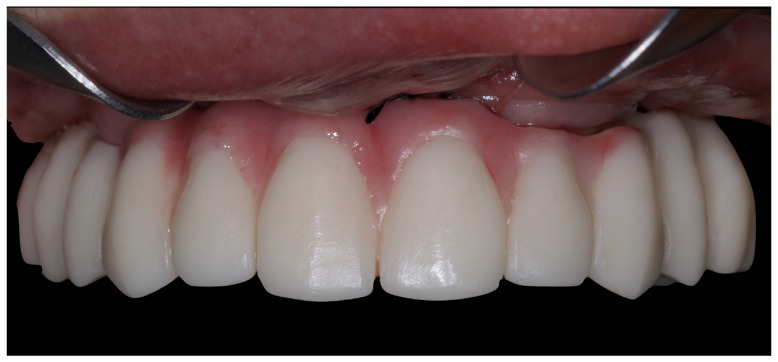
Upper immediate FCD.

In the dental laboratory, all STL files (PIC files, preoperative soft tissues scan, and postoperative soft tissues scan) were aligned to obtain the virtual definitive models using a software (Exocad 2.4 Plovdiv; Exocad GmbH) (Figure 7). The virtual provisional FCDs’ predesign was aligned with the definitive models and modified according to post-surgical soft tissue form. The provisional screw-retained FCDs were milled (Figure 8), and placed six hours after the surgery (Figure 9). Polyoxymethylene resin (POM), also known as polyacetal, was the selected material for the immediate prostheses. Before the insertion of the immediate prostheses the passive fit was verified intraorally, and both prostheses were seated without resistance. All prosthetic screws were tightened to 15 Ncm, according to manufacturer’s recommendations. After prostheses insertion, the occlusion was rechecked (mutually protected occlusion type was achieved), and then all screw’s access channels were sealed with light-curing temporary filling material (Clip Flow; VOCO GmbH, Cuxhaven, Germany).

**Figure 10:**
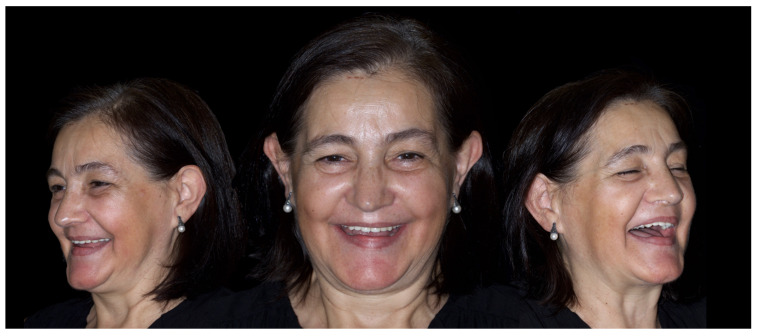
Extraoral aspects of the patient one week after surgery.

**Figure 11:**
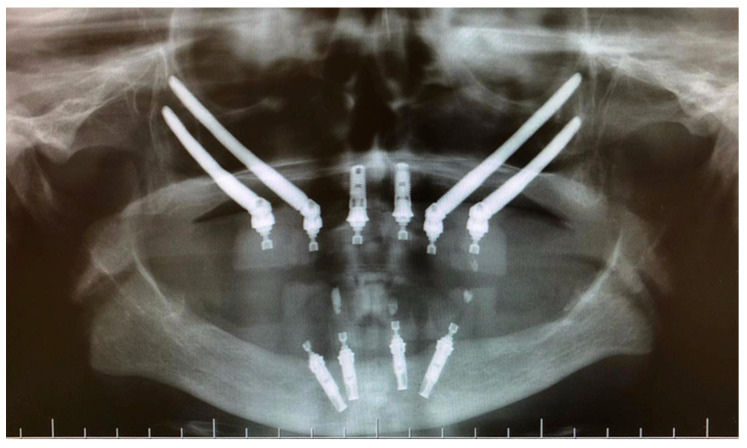
Panoramic radiograph.

One week later, the patient was recalled for the first follow-up (Figure 10). Clinical re-evaluation was performed and a panoramic radiograph was taken (Figure 11); the patient complained less, and the provisional prosthesis showed good esthetic and functional behaviours. 

**Figure 12:**
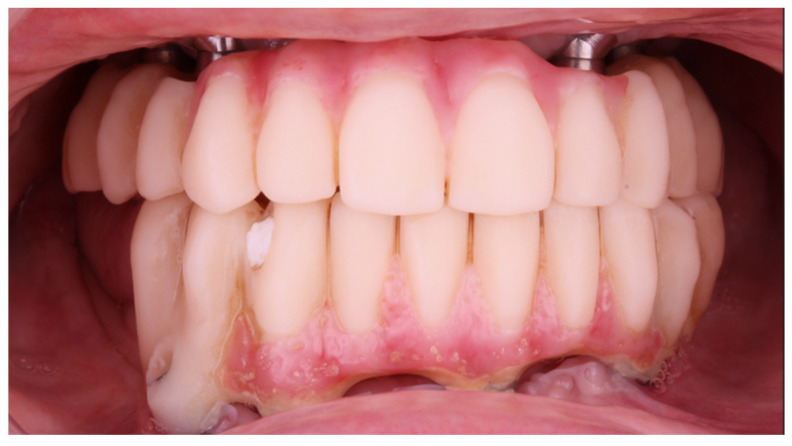
Intraoral view of the provisional FCDs at six-months follow-up.

The patient was re-evaluated after three and six months, and the provisional maxillary prosthesis showed the same results as the previous re-evaluation; except, there a slight increase in the transitional space between the soft tissue and the provisional prosthesis (Figure 12). This was not considered an issue because it is a normal consequence of the soft tissue healing process and it would be corrected with the definitive prosthesis. 

At the end of the complete implant osteointegration time (six months), all functional and esthetic parameters were re-evaluated. The provisional prostheses had some esthetic limitations: the color did not match the patient’s desire, there was a little deviation in dental midline, and the patient also wanted to show a little more teeth exposure at the rest and smile position.

**Figure 13:**
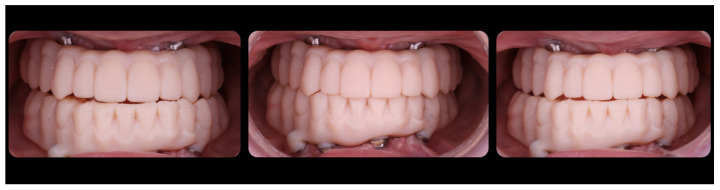
Functional analysis of the prototype.

The provisional prostheses were removed, all transmucosal abutments screws were re-tightened to 30 Ncm, a new soft tissue scan was obtained with the IOS, both prostheses were inserted and scanned, and the intermaxillary relation was also scanned. All STL files, lab notes, and photo series were sent to the technician to design and fabricate the definitive prostheses. The prototype prostheses were 3D printed using polymethylmethacrylate (PMMA), and a try-in was carried out. The try-in showed excellent esthetic and functional results (Figure 13), all the immediate prostheses’ limitations were corrected, and the patient was satisfied. In the lab (virtually), a cut-back of 2 mm was carried out in the final virtual design to reserve space for veneering materials. In addition, laser sintering cobalt-chromium frameworks were fabricated, and to achieve the maximum passivity, all framework’s connections were milled.

**Figure 14:**
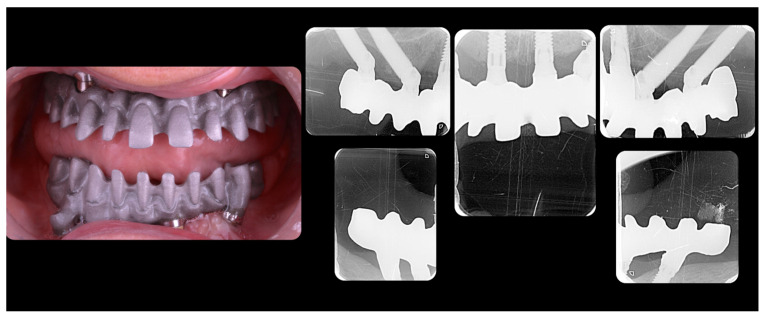
Frameworks try-in.

The patient was recalled and the frameworks tray-in was carried out (Figure 9). Both frameworks were seated comfortably without any noted frictions, showing a good passive fit in the intraoral tests (the alternative fingers technique, direct vision, and intraoral Sheffield’s test) [21]. Moreover, in the radiographic examination, neither framework showed any radiographic misfit (Figure 14). Both frameworks were resent to the technician for veneering with a composite resin. 

One week later, both definitive screw-retained FCDs were tested in the mouth prior to insertion for esthetics and function. Maintenance techniques were re-explained to the patient, and the review visits were planned for every 6 months. The FCDs were examined every 6 months up 2 years after the end of the treatment. Mechanical, esthetic, or biological complications were recorded. Periapical radiographs and clinical photographs were obtained at each follow-up. 

## 3. Results

A patient with completely edentulous jaws received two srew-retained FCDs. The maxillary arch received six implants and the mandibular arch received four implants. The implants and the prostheses survival and success rate at 2 years was 100%.

**Figure 15:**
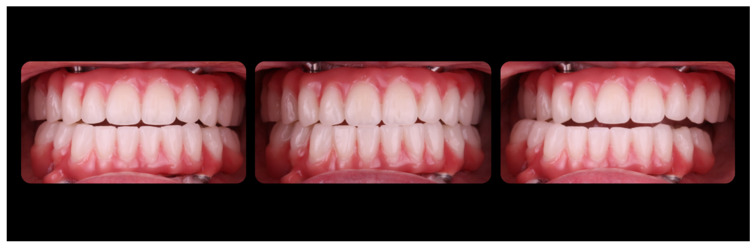
Intraoral view of the FCDs 1-year after insertion.

The patient was examined at a 1 week, 6-, 12-, 18-, and 24-months follow-up and no technical complications, such as prosthesis fracture, screw loosening, or other complications, were observed. (Figure 15) In the same way, no biological complications were observed. The soft peri-implant tissues were healthy, the implants’ osseointegration were found to be normal, and no marginal bone loss was observed in the control radiographs taken at every recall. 

**Figure 16:**
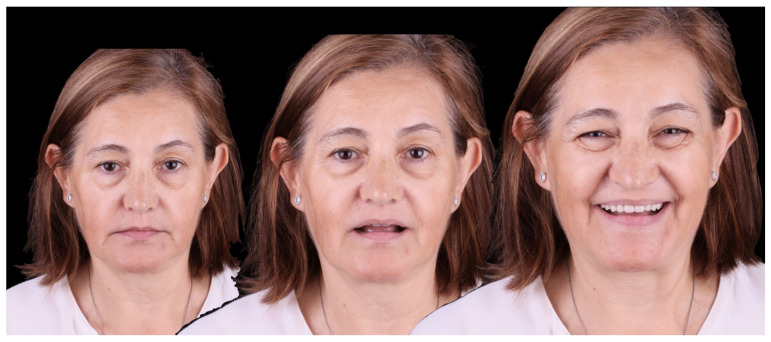
Aspect of the patient at the 2-year follow-up evaluation.

Esthetics and function remained stable over the follow-up period (Figure 16). The patient felt satisfied with the dual scan technique used and expressed that it was a comfortable experience.

## 4. Discussion

In the pre-maxilla, the alveolar bone is rapidly re-contoured after the loss of teeth. In the first year, the volume decreases by approximately 25%, and in the first 3 years the width decreases by 40 to 60%. In the posterior areas of the maxilla, the rate of bone loss is greater than in the anterior sectors, due to the fact that the posterior dimension of the crest is twice as large as the anterior one [22]. In these situations, zygomatic implants have been offered as a treatment option for prosthetic rehabilitations to avoid the possible complications associated with bone grafting procedures and reduce the time of treatment.

In the presented case, following 24 months of follow-up evaluation, no biological or mechanical complications were observed in the implants or in the prostheses. The success of the immediate loading protocol was influenced by different factors, mainly divided into four categories: surgery-related factors, host-related factors, implant-related factors, and occlusion-related factors [23]. Some complications have been reported in the literature, such as implant failure, prosthesis failure, implant fracture, prosthetic and abutment screw loosening, soft tissue inflammation and sinusitis, speech complications, and hygiene difficulties [7,8,9,10,11,12,13]. In general, the complications ass immediately loaded zygomatic implants are rare, and most of them could be resolved easily in the clinic. The alveolar bone guide was selected to avoid possible biological complications such as sinusitis [4].

The type of prosthetic material is an essential factor in the longevity and success of the immediate prosthesis. Menini et al. suggested that the use of a rigid framework in full-arch FCDs provides a better load distribution that decreases stress at the implant level [24]. In this report, POM resin was the selected material; a thermoplastic polymer, showing good physical, chemical, and mechanical properties such as its high rigidity, high dimensional stability, high resistance to bending, abrasion resistance, and biocompatibility, with the only inconvenience of being less esthetic than other resin materials [25,26]. Another great advantage of the screw-retained provisional FCDs fabricated with POM resin is that they can be directly connected to implant or abutment platforms without a titanium interface, which leads to the elimination of any unexpected errors generated by titanium cylinders cementation and remove their cost.

For immediate screw-retained FCDs, tension-free seating is required to prevent any undesired force to the recently placed implants. In the present study, CAD/CAM technology was implicated to improve the precision and to reduce the working time. The CAD/CAM-fabricated prosthesis requires a precise virtual model that can be obtained from the indirect scanning of a plaster cast obtained using the conventional impression technique, and, although the passive fit of the framework might be achieved, the use of this technique in immediate loading protocols could be harmful to the immediately placed implants. Moreover, it will increase chair-side and laboratory working times, and it is not considered as a complete digital workflow. The virtual model can be also obtained by using an IOS in a complete digital workflow. However, until now, the accuracy of the IOSs is still insufficient in the case of full mouth implant rehabilitation [27]. The accuracy of the stereophotogrammetry technique is comparable to the conventional impression technique, and the discrepancy is within the clinically accepted level, as previously reported [18,19,28,29,30,31]. However, previous clinical studies are scarce. Pradíes et al. published a case series, explaining sterophotogrammetric technology, and they concluded that the mentioned technology is viable and accurate for creating implant impressions [32]. Pozzi et al. clinically evaluated the accuracy of IOS and stererophotogrammetry full arch implant impression, and they mentioned that the stereophotogrammetry impression technique shows a higher linear and angular accuracy than IOS impression technique [33]. In a recent clinical study, Zhang et al. [34] evaluated the accuracy of photogrammetric imaging compared to conventional splinted impressions, reporting that the accuracy was within the acceptable range of error, and that the interimplant angulations and the jaw had no effect on accuracy. However, only two of the available stereophotogrammetry systems have been analyzed in the studies, with differences in the accuracy between them [30]. Stereophotogrammetry has the additional advantage over the conventional and IOS impressions that no new records of the implants’ 3D locations are necessary to fabricate the definitive prostheses [25].

The only disadvantage of the stereophotogrammetry is that it only records the 3D position of implants without registering the soft tissues. This limitation can be overcome by scanning the soft tissue with or without scan bodies using an IOS, or indirectly by scanning of plaster cast with an extraoral scanner or negatively scanning an alginate impression with an IOSs. In the present report, the soft tissue was scanned using an IOS. Besides the rapidity and the affectivity of the stereophotogrammetry, it is also not affected by the patient’s or clinician’s movements. In an in vivo study with a one-year follow-up published by Peñarrocha-Diago et al. [31], comparing the stereophotogrammetry impression technique and the conventional impression technique, it was shown that, with stereophotogrammetry, the patient’s and clinician’s satisfaction was improved and the working time was reduced, and, in the same study, the authors did not find any significant differences in terms of implant success rate, implant survival, marginal bone loss, or prosthesis survival between the two experimental groups. 

The combination of an IOS and the stereophogrammetry system used in this study obtained a precise fit of both provisional and definitive FCDs, avoiding the cumulative errors associated with the conventional impression technique, and allowing the fabrication of the prostheses in a complete digital workflow. Patient satisfaction was positive at all evaluations during the follow-up period. 

Previous clinical studies evaluating the viability of the stereophotogrammetry technique for full arch implant impression are very limited. Nevertheless, randomized clinical trials with long-term evaluation period are necessary for reliable results and to be able to extend the technique to the daily clinic. 

## 5. Conclusions

The dual-scan technique presented in this report might be an effective and reliable alternative for the fabrication of immediate and definitive screw-retained FCDs, using a complete digital workflow, especially in complex cases such as zygomatic implants. The time of scanning and fabrication was reduced, and the clinician’s and patient’s satisfaction were improved. Further clinical studies are necessary to ensure the reliability of the use of the stereophotogrammetry technique in intraoral implant scanning.

## Data Availability

Data presented in the manuscript are contained within the article.
